# Mental health and weight regain after bariatric surgery: associations between weight regain and psychiatric and eating-related comorbidities

**DOI:** 10.20945/2359-4292-2023-0208

**Published:** 2024-07-12

**Authors:** Maria Francisca F. P. Mauro, Marcelo Papelbaum, Marco Antônio Alves Brasil, João Regis Ivar Carneiro, Ronir Raggio Luiz, João C. Hiluy, José Carlos Appolinario

**Affiliations:** 1 Grupo de Obesidade e Transtornos Alimentares Instituto de Psiquiatria Universidade Federal do Rio de Janeiro Rio de Janeiro RJ Brasil Grupo de Obesidade e Transtornos Alimentares, Instituto de Psiquiatria, Universidade Federal do Rio de Janeiro, Rio de Janeiro, RJ, Brasil; 2 Hospital Universitário Clementino Fraga Filho Universidade Federal do Rio de Janeiro Rio de Janeiro RJ Brasil Hospital Universitário Clementino Fraga Filho, Universidade Federal do Rio de Janeiro, Rio de Janeiro, RJ, Brasil; 3 Instituto de Estudos de Saúde Pública Universidade Federal do Rio de Janeiro Rio de Janeiro RJ Brasil Instituto de Estudos de Saúde Pública, Universidade Federal do Rio de Janeiro, Rio de Janeiro, RJ, Brasil

**Keywords:** Weight gain, weight recurrence, psychiatric comorbidity, binge eating, eating behavior, impulsivity

## Abstract

**Objective:**

Weight regain is a common outcome of weight loss interventions. Mental health-related comorbidities, among other factors, can mediate weight regain regardless of the implemented treatment modality. This study explores whether postoperative psychopathological comorbidities are associated with weight regain after bariatric surgery.

**Subjects and methods:**

This cross-sectional study recruited 90 outpatients who underwent Roux-en-Y gastric bypass surgery. Anthropometric measurements were collected retrospectively from medical charts. The Structured Clinical Interview for Diagnostic and Statistical Manual of Mental Disorder-IV (DSM-IV) Axis I Disorders (SCID-I) was applied to evaluate psychiatry diagnoses. Validated self-report instruments were used to assess depression, anxiety, alcohol use, impulsivity, binge eating, and body image dissatisfaction. Weight regain was defined as a ≥20% increase from the maximum weight lost. Level of evidence: Level III, cross-sectional study based on a well-designed study.

**Results:**

Overall, 55.6% of participants experienced weight regain. Notably, mental disorders such as current binge-eating disorder and lifetime diagnoses including bulimia nervosa, alcohol abuse/dependence, and obsessive-compulsive disorder were significantly associated with weight regain. However, controlled analysis found that, for mental disorders, only current binge-eating disorder (odds ratio [OR] 6.3, 95% confidence interval [CI] 1.26-31.06, p = 0.024) remained associated with weight regain. Eating-related psychopathologies also associated with weight regain included binge eating (d = 0.55; p = 0.013), eating disinhibition (d = 0.76; p = 0.001), higher hunger levels (d = 0.39; p = 0.004), and non-planning trait impulsivity (d = 0.69; p = 0.0001).

**Conclusion:**

Postoperative presence of psychopathological comorbidities, such as eating psychopathology and trait impulsivity, were associated with weight regain after bariatric surgery. These findings highlight the importance of addressing mental health in individuals experiencing postsurgical weight regain.

## INTRODUCTION

Bariatric surgery is widely recognized as the standard treatment for severe obesity (1). Numerous studies have consistently demonstrated the benefits of bariatric surgery, including significant weight loss, improved control or resolution of clinical comorbidities, and enhanced quality of life (2). However, it is important to acknowledge that weight regain can occur after this type of surgery, with reported incidences ranging from 9% to 91% (3). The variability in reported rates is influenced by the differing definitions of weight regain across various studies and the absence of a standardized definition, making it challenging to address this outcome consistently (2,3). Some evidence suggests the use of the percentage of the maximum amount of weight lost from the lowest postoperative weight after surgery as a standardized measure to assess weight regain (2-5). A reduction exceeding 20% from this parameter is considered a possible marker of significant weight recidivism (2). Addressing this evidence-based categorization in clinical investigations could contribute to improving our understanding of this outcome following bariatric surgery.

Several risk factors have been identified as potentially contributing to weight regain after bariatric surgery, including the natural course of obesity, surgical-related factors, behavioral aspects, and clinical variables (1). Significant weight regain following bariatric surgery may lead to the deterioration of metabolic improvements achieved through the procedure, including increased insulin resistance, reemergence of diabetes, worsening nonalcoholic fatty liver disease, and recurrence of obesity-related comorbidities (2,6,7). Moreover, a growing body of evidence suggests that psychopathological comorbidities may contribute to poor bariatric surgery outcomes (4).

There is robust evidence showing an increase in poor mental health in individuals with obesity compared with those with a normal body mass index (BMI) (8), indicating a greater association between clustering of physical multimorbidity and mental health in individuals with obesity (8,9). Studies reporting such evidence indicate common pathways linking obesity to mental disorders, including genetic influence, obesity biomarkers (insulin resistance, inflammatory processes, oxidative damage), environmental factors (alcohol use, physical inactivity, socioeconomic status), and distress (8-11).

The frequency of comorbid mental disorders in individuals with severe obesity seeking bariatric surgery is higher than that in the general population (12). Previous studies have focused on the relationship between psychopathological comorbidities prior to bariatric surgery and weight regain (5,13-17). Most of these studies have shown that preoperative psychopathology did not result in a greater degree of weight regain (18). However, the course of psychiatric comorbidities after bariatric surgery remains unclear. Some prospective studies have reported a decline (14,15,19) in comorbid mental disorders after bariatric surgery compared with the preoperative period, while others have reported that the incidence remained stable or increased in the long term after surgery (14,16). When considering eating psychopathology specifically, a subgroup of individuals may experience a reduction in their eating psychopathological symptoms after bariatric surgery (5).

Some studies have shown that postoperative psychopathology, such as depressive symptoms (20) and attentional impulsivity (21), are positively associated with weight regain (13) and deterioration in health-related quality of life (22,23). A meta-analysis published in 2019 found that postoperative eating psychopathologies, such as binge eating, loss of control while eating, and grazing, are associated with a greater risk (odds ratio [OR] 2.2) of weight regain (13). To summarize, the available evidence suggests that individuals undergoing bariatric surgery are more susceptible to mental disorders both before and after surgery (12,24).

Mental impairment has a complex nature and can be conceptualized as diagnostic categories or clusters of psychopathological symptoms (16). While the categorical perspective determines the presence or absence of a specific mental disorder, the dimensional perspective considers the existence of psychopathology along a continuum of normal to severe (25). This difference in assessment could contribute to the superior ability of the dimensional approach in detecting subtle mental impairment phenomena (16). Therefore, an analysis that considers both parameters could be more sensitive and accurate for evaluating psychopathological manifestations, especially among individuals undergoing bariatric surgery, due to the anatomic and metabolic changes induced by the procedure (26).

Given these observations, the present study used a structured assessment methodology to investigate the interplay between postoperative psychopathology (categorical and dimensional) and weight regain after bariatric surgery. The aim of the study was to investigate the relationship between weight regain and psychopathology among patients who had undergone Roux-en-Y gastric bypass (RYGB) and had a minimum postoperative period of 18 months. This time frame was chosen based on the weight trajectory following RYGB and the occurrence of weight regain.

## MATERIALS AND METHODS

### Participants

The present study used a convenience sample and was conducted at the Bariatric Surgery Program of the Clementino Fraga Filho University Hospital at the Federal University of Rio de Janeiro (UFRJ). The inclusion criteria were participants who (A) had undergone RYGB, (B) were 18 years or older, (C) were of any sex, and (D) had a minimum postoperative period of 18 months. The exclusion criteria were (A) untreated endocrine disease, (B) pregnancy, or (C) corticosteroid use.

The institutional review board approved the study protocol (CAAE number: 53712516.6.0000.5257). All participants provided written informed consent before undergoing any study procedure.

### Procedures and assessments

**Figure 1:**
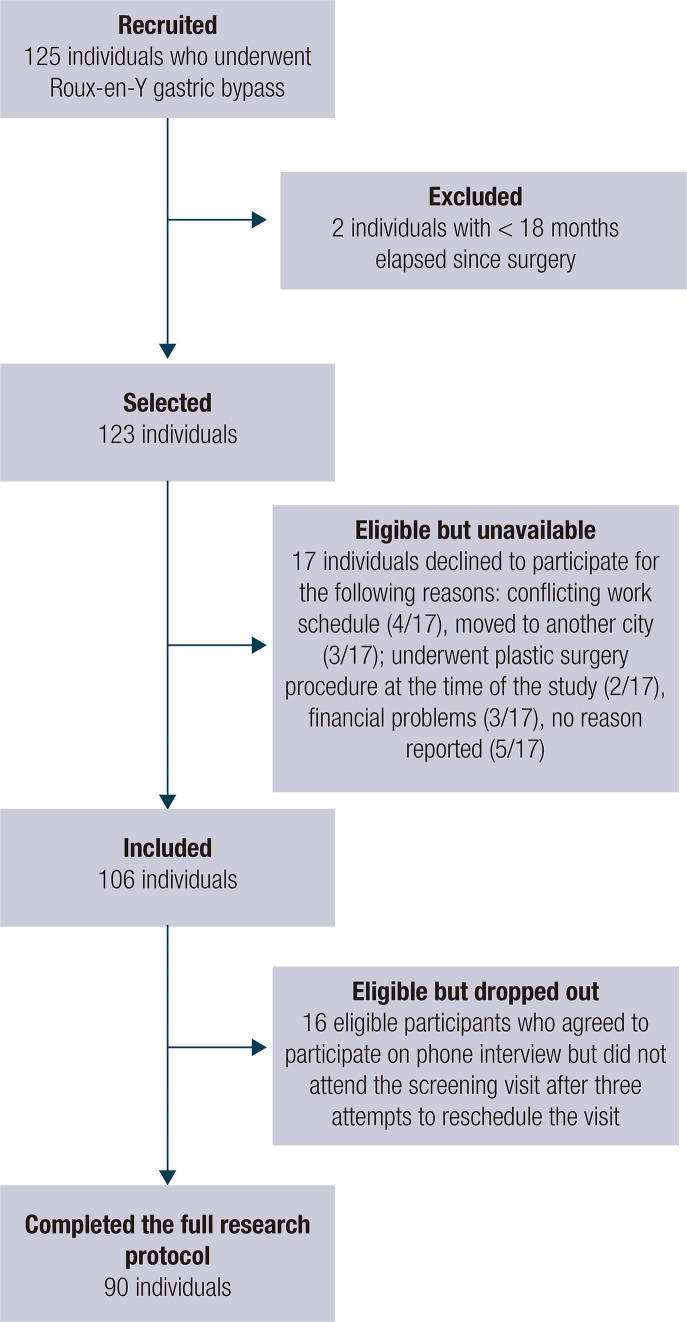
Flow chart of the participants' selection.

A total of 125 individuals who had undergone RYGB surgery were sequentially contacted and invited to participate during their appointments at the hospital or via telephone between September 2016 and October 2018. Of all individuals contacted, 90 (72%) completed the full research protocol (Figure 1).

The participants were evaluated by psychiatrists (MFFPM and JCH) experienced and trained in the use of psychiatric assessment instruments. During the face-to-face interview, the following variables were collected:

- Demographics.
- Clinical variables. We collected data on psychiatric medication use, including the names of the medications and their frequency of utilization. Subsequently, we classified these medications into categories, which included antidepressants, antianxiety drugs, anticonvulsants, antipsychotics, medications for mania/mood stabilizers, and central nervous system stimulants (14).
- Occurrence of mental disorders (current and lifetime) and psychopathological symptoms (self-report scales), assessed using a structured interview.
- Weight, collected retrospectively from the participants’ medical charts and measured at admission in the surgical unit. We collected the following parameters: preoperative weight, measured on surgery day, and lowest postoperative weight. The current weight was measured during the face-to-face interview using a balance beam scale and with the participants without shoes and wearing light clothes. In the cases we did not find reliable information regarding the lowest postoperative weight in the medical charts, we considered the postoperative lowest weight reported by the participant.
- Height, collected from the participants’ medical charts and measured at admission in the surgical unit.

To assess the impact of time in our analysis, we created groups based on the postoperative time in months (short/medium follow-up group: 18-59 months; longer follow-up group: ≥60 months) (27). The time required to achieve maximum weight loss was calculated as the difference between the date of the surgery (month/year) and the date of the minimum postoperative weight (month/year). Subsequently, groups for the time to achieve maximum weight loss were defined as 0–24 months and >24 months (2).

### Mental health assessment

#### Mental disorders (categorical)

Mental disorders were assessed using the Structured Clinical Interview for Diagnostic and Statistical Manual of Mental Disorders, Fourth Edition (DSM-IV) Axis I Disorders (SCID-I), a semi-structured interview tool used to classify mental disorders. The SCID-I Patient Edition is a well-validated instrument for the assessment of current and lifetime diagnoses of mental disorders. The SCID-I version used had been translated into Portuguese and adapted to the Brazilian population (28). The eating disorders section was based on the classification criteria established by the Diagnostic and Statistical Manual of Mental Disorders, Fifth Edition (DSM-5) (29).

Specific psychopathologies were evaluated using validated self-report questionnaires during in-person assessment to determine current psychopathology.

#### General psychopathology (dimensional)

For general psychopathology, participants completed the Beck Depression Inventory (BDI), which contains 21 self-rated items measuring the intensity of depressive symptoms in the past 2 weeks (30). Trait impulsivity was assessed using the Barratt Impulsiveness Scale (BIS-11), a self-reported 30-item Likert scale that measures impulsiveness in different domains, namely, motor impulsiveness (incoherent action in a given context), attentional impulsiveness (inability to focus attention or concentrate), and non-planning impulsiveness (decrease in orientation toward the future); higher scores indicate greater severity of impulsiveness (31).

#### Eating psychopathology (dimensional)

Eating psychopathology was assessed using the Binge Eating Scale (BES), a 16-item Likert scale measuring the severity of binge eating (≥27 = severe; 18-26 = moderate; ≤17 = no binge eating) (32). Additionally, the Three-Factor Eating Questionnaire (TFEQ) (33), a 51-item scale, was applied to measure eating behavior in three domains: restriction (restricting food intake to control body weight), disinhibition (tendency toward overeating and eating in response to external food stimuli), and hunger (eating episodes elicited by hunger signals) (34). Finally, body shape concerns were evaluated using the Body Shape Questionnaire (BSQ), a 34-item Likert scale that quantifies concerns about body image in the past 4 weeks (35). The self-report instruments used in this study had been validated in the context of the Brazilian population (30-32,34,35).

## Weight regain

Weight regain was calculated as the percentage of weight regained after surgery, considering the difference between the current weight and the lowest weight after surgery, relative to the difference between the weight measured before and after surgery: [100*(current weight – lowest postoperative weight)] / (preoperative weight – lowest postoperative weight)], in which the lowest postoperative weight was the minimum postoperative weight achieved after the RYGB (2). Participants were stratified into groups based on a weight regain cutoff value of ≥20%, per this calculation. Thus, patients with weight regain ˂20% were classified into the non-weight regain group (“NWR group”), while those with weight regain ≥20% were included in the weight regain group (“WR group”) (1).

## Weight loss

Weight loss after surgery was calculated as a percentage of preoperative weight and BMI variation. We applied the calculation of the total weight loss using the formula: [100*(preoperative weight - current weight) / preoperative weight] (27). Subsequently, we defined groups based on total weight loss <20% and ≥20% to examine insufficient weight loss (3). The weight loss measure of percent excess weight loss was also calculated. The calculation was based on the formula: [(preoperative weight - current weight) / (preoperative weight - ideal weight)]. We then defined groups based on percent excess weight loss <50% and ≥50% to investigate a surgical nonresponse (3). A BMI change was calculated as follows: [preoperative BMI - current postoperative BMI] (3,27).

## Statistical analysis

Descriptive analyses were performed to summarize the demographic data, anthropometric measurements, and clinical variables (frequencies and percentages for categorical variables and medians with minimum–maximum values for continuous variables). Our dependent variable was weight regain at the established cutoff (≥20% from the maximum weight loss). Independent variables included clinical variables (psychiatric medication use, postoperative time as a continuous variable in months, postoperative time groups based on short/medium follow-up [18-59 months], and longer follow-up [participants included if they had ≥60 months of postoperative time]), mental disorders (lifetime and current), and psychopathological symptoms (binge eating measured by the BES, eating behavior assessed by TFEQ, shape concerns evaluated by BSQ, depressive symptoms assessed by BDI, and trait impulsivity measured by BIS-11).

The Mann-Whitney U test for nonparametric data was performed to compare continuous clinical variables, psychopathological symptoms measured by the self-report questionnaires, and the defined weight regain cutoff. The effect size was calculated based on the median differences between the groups, with the standard deviation in the variance of this calculation. Subsequently, the statistical significance of the effect size was calculated using Cohen’s d statistic. We adopted Cohen’s suggestion that d values of 0.2, 0.5, and 0.8 could be considered to represent a small, medium, and large effect size, respectively (36). The frequencies of mental disorders in the weight regain categories were compared using the chi-squared test.

We conducted a stepwise automatic logistic regression analysis controlling for confounding variables in the association between mental disorders and weight regain. First, covariates (sex, postoperative time groups, postoperative time to the maximum weight loss groups, lifetime obsessive-compulsive disorder, lifetime alcohol abuse/dependence, lifetime bulimia nervosa, and current binge-eating disorder) were introduced into the model. Second, only covariates that remained statistically significant in the equation were described, and their ORs were calculated along with 95% confidence intervals (CIs).

The data were analyzed using the Statistical Package for Social Sciences (SPSS), Version 21 (IBM Corp., NY, USA). Statistical significance was set at p < 0.05.

## RESULTS

### Participants’ characteristics

The characteristics of the study population were as follows: median age, 48 years (range 26-76 years); women, 73 (81.1%); median preoperative BMI, 50 kg/m^2^ (range 36.2-77.5 kg/m^2^); and median current postoperative BMI, 33.9 kg/m^2^ (range 23-56.1 kg/m^2^). The median postoperative follow-up was 130.5 months (range 19-217 months). Participants exhibited a median percentage weight regain of 21.6% (range 0-111.8%). Out of the 90 participants, 36 (40%) were using psychiatric medications, which were categorized as follows: anticonvulsants (1/90), antidepressants (18/90), antianxiety drugs (14/90), antipsychotics (0/90), medications for mania/mood stabilizers (0/90), and central nervous system stimulants (1/90). Comparison of demographic and surgical parameters between the NWR and WR groups revealed statistically significant differences in sex at birth. In our sample, 73 participants (81.1%) had a postoperative follow-up duration that exceeded 60 months. When we compared the WR and NWR groups concerning the cutoff of 60 months, we observed that participants in the WR group had a significantly longer postoperative time (WR: 46 participants [92%] *versus* NWR: 27 participants [67.5%]; p = 0.003). Regarding the subgroups based on time to achieve the lowest postoperative weight, 64 participants (71.1%) reached their lowest weight within 24 months or less. Participants in the WR group, based on the cutoff of 24 months, achieved their lowest weight in a shorter time frame compared with those in the NWR group (WR: 40 participants [80%] vs. NWR: 24 participants [60%]; p = 0.038). Additionally, significant differences between the NWR and WR groups were observed in terms of weight loss outcome of BMI change (Table 1).

**Table 1:** Demographic and clinical characteristics of individuals with and without weight regain after Roux-en-Y gastric bypass

		Groups	
	NWR (n=40)	WR (n=50)	P value*
Age, years – median (min-max)	48.5 (26-76)	48 (29-76)	0.47
Birth sex – n (%)			
Female	37 (92.5)	36 (72)	**0.014**
Male	3 (7.5)	14 (28)	
Race – n (%)			
White	17 (42.5)	30 (60)	0.099
Not white	23 (57.5)	20 (40)	
Marital status – n (%)			
Not married	18 (45)	22 (44)	0.92
Married or in common-law marriage	22 (55)	28 (56)	
Educational level – n (%)			
0-8 years	9 (22.5)	15 (30)	0.67
9-13 years	19 (47.5)	23 (46)	
>13 years	12 (30)	12 (24)	
Psychiatric medication use	13 (32.5)	23 (55.6)	0.19
Preoperative BMI – median (min-max)	49.8 (36.7-71.8)	50.3 (36.2-77.5)	0.71
Current BMI – median (min-max)	32.2 (24.2-48.9)	36.5 (23-56.1)	**<0.001**
Change in BMI Δ – median (min-max)	17.8 (8.6-38.5)	13 (-2.7-27.1)	**<0.001**
Postoperative time, months – median (min-max)	129 (19-217)	132 (29-182)	0.48
Postoperative time to the maximum weight loss, months – median (min-max)	19 (3-168)	13.5 (3-72)	0.12
Groups based on < 50% of EWL)	8 (20)	9 (18.4)	0.84
EWL (%) – median (min-max)	61.1 (31.2-125.1)	70.8 (26.2-162)	0.77
Groups based on <20% of TWL)	1 (2.5)	3 (6)	0.42
TWL (%) – median (min-max)	68.2 (16.3-139)	68 (4.2-175)	0.4
Weight regain from the maximum weight loss, % –median (min-max)	11.5 (0-19.5)	33.6 (20-111.8)	**0.0001**

*The analysis was performed using the chi-square test for categorical variables and the Mann-Whitney U test for continuous variables. Significance was defined by a p value ≤ 0.05.

Abbreviations: Δ, delta; %, percentage; BMI, body mass index, calculated as weight divided by the square of height; EWL; excess weight loss; NWR, no weight regain group; TWL, total weight loss; max, maximum; min, minimum; n, number; WR, weight regain group.

In the sample (n = 90), 57.7% (n = 52) and 76.6% (n = 69) participants had a current and lifetime mental disorder, respectively. Mood disorders were the most frequent diagnostic category in the entire sample, with 41.1% (n = 37) of participants presenting a lifetime and 30% (n = 27) a current mood disorder. Lifetime major depressive disorder was the most prevalent diagnosis, observed in 41.1% (n = 37) participants, followed by current depressive episode, observed in 28.8% (n = 26) of them. Of note, 60% (n = 54) of participants displayed some type of anxiety disorder. Specifically, 53.3% (n = 48) of participants met diagnostic criteria for a lifetime anxiety disorder, while 38.9% (n = 35) had a current anxiety disorder. Lifetime specific phobia had the highest prevalence in the overall sample (23.3%; n = 21), followed by lifetime social phobia (16.6%; n = 15), current generalized anxiety disorder (13.3%; n = 13), and lifetime generalized anxiety disorder (4.4%; n = 4). Additionally, 27.7% (n = 25) of participants met diagnostic criteria for lifetime post-traumatic stress disorder and 14.1% (n = 13) for current post-traumatic stress disorder. Lifetime alcohol abuse was present in 13.3% (n =12) of the sample, while a diagnosis of current alcohol abuse was present in 11.1% (n = 10). Lifetime binge-eating disorder was the second most prevalent mental disorder, occurring in 36.6% (n = 33) of participants.

### Association between mental disorders and weight regain

Comparing the groups, when the mental disorders were pooled in a class analysis for the presence of any lifetime or current mental disorder, the WR group (n = 50) exhibited a prevalence of 86% (n = 43) compared with 72.5% (n = 29) in the NWR group (n = 40). However, these differences between the NWR and WR groups were only significant for specific mental disorders, namely, alcohol abuse/dependence (5% *versus* 20%, respectively, p = 0.03), obsessive-compulsive disorder (2.5% *versus* 14%, respectively, p = 0.05), current binge-eating disorder (7.5% *versus* 24%, respectively, p = 0.03), and lifetime bulimia nervosa (0 *versus* 12%, respectively, p = 0.02). Differences in prevalence of mental disorders between the groups are shown in Table 2.

**Table 2:** Comparison of lifetime and current prevalence of mental disorders in individuals with and without weight regain after Roux-en-Y gastric bypass

Mental Disorders (DSM-IV*)	Lifetime Prevalence		P value	Current Prevalence		P value
	NWR n=40 n (%)	WR n=50 n (%)		NWR n=40 n (%)	WR n=50 n (%)	
Mood disorders						
Major depressive disorder	13 (32.5)	24 (48)	0.13	9 (22.5)	17 (34)	0.23
Dysthymia	0	0	N/A	0	1 (2)	0.36
Bipolar disorder	0	1 (2)	0.36	2 (5)	0	0.11
Anxiety disorders						
Social phobia	7 (17.5)	8 (16)	0.85	7 (17.5)	7 (14)	0.64
Specific phobia	10 (25)	11 (22)	0.73	N/A	N/A	N/A
Generalized anxiety disorder	1 (2.5)	3 (6)	0.42	3 (7.5)	9 (18)	0.14
Panic disorder	2 (5)	6 (12)	0.24	5 (12.5)	5 (10)	0.7
Agoraphobia	2 (5)	8 (16)	0.09	5 (12.5)	9 (18)	0.47
Post-traumatic stress disorder	10 (25)	15 (30)	0.6	7 (17.5)	6 (12)	0.46
Obsessive-compulsive disorder	1 (2.5)	7 (14)	**0.05**	2 (5)	4 (8)	0.57
Body dysmorphic disorder	2 (5)	3 (6)	0.83	N/A	N/A	N/A
Somatoform disorder	2 (5)	4 (8)	0.57	N/A	N/A	N/A
Substance use disorder						
Alcohol abuse	2 (5)	10 (20)	**0.03**	4 (10)	6 (12)	0.76
Alcohol dependence	2 (5)	10 (20)	**0.03**	2 (5)	4 (8)	0.57
Other substance abuse/dependence	5 (12.5)	7 (14)	0.83	1 (2.5)	1 (2)	0.87
Eating disorders*						
Anorexia nervosa	0	1 (2)	0.36	0	0	N/A
Bulimia nervosa	0	6 (12)	**0.02**	1 (2.5)	0	0.26
Binge-eating disorder	14 (35)	19 (38)	0.76	3 (7.5)	12 (24)	**0.03**

The analysis was performed using the chi-square test. Significance was defined by a p value ≤ 0.05. *The diagnosis of eating disorders was based on DSM-5 criteria.

Abbreviations: N/A, not available; NWR, no weight regain; WR, weight regain.

The controlled analysis examining the association between the WR cutoff and mental disorders revealed that only current binge-eating disorder remained significantly associated with the dependent variable (OR 6.3, 95% CI 1.26-31.06; p = 0.024), as shown in Table 3.

**Table 3:** Multivariate logistic regression analysis* controlling for confounding variables (postoperative time, postoperative time to maximum weight loss, and psychiatric comorbidity) in the association between mental disorders and weight regain after bariatric surgery

Covariates	OR (95% CI)	P value
Postoperative time groups		
Group 1 (18-59 months)	1	
Group 2 (60-217 months)	7.2 (1.86-27.52)	0.004
Postoperative time to maximum weight loss groups		
Group 1 (0-24 months)	5.3 (1.69-16.91)	0.004
Group 2 (>24 months)	1	
Birth sex		
Female	1	
Male	6.4 (1.31-31.18)	0.022
Binge-eating disorder**		
Yes	6.3 (1.26-31.06)	0.024
No	1	

Dependent variable: weight regain ≥ 20% from the maximum weight lost. *Method stepwise: In the first step, we introduced each covariate into the regression model, along with the dependent variable. The covariates were sex, postoperative time groups, postoperative time to maximum weight loss groups, lifetime obsessive-compulsive disorder, lifetime alcohol abuse/dependence, lifetime bulimia nervosa, and current binge-eating disorder. In the second step, we included only the covariates that remained statistically significant in the equation model after the first step. **Current diagnostic.

Abbreviations: 95% CI, 95% confidence interval; OR, odds ratio.

### Association between psychopathology and weight regain

We found significant differences in psychopathology intensity between groups in terms of binge eating (BES), eating behaviors dimensions of disinhibition, restriction, and hunger (TEFQ), and non-planning impulsivity (BIS-11). The results for the comparison between the level of psychopathology and the weight regain groups are shown in Table 4. The effect size for the psychopathology degree and TFEQ restriction (d = -0.60) and hunger (d = 0.39) was small. We found a medium effect size of the BES score (d = 0.55), TFEQ disinhibition (d = 0.76), and BIS-11 domain of non-planning impulsivity (d = 0.69) for weight regain.

**Table 4:** Comparison of general and eating psychopathologies in individuals with and without weight regain after Roux-en-Y gastric bypass

General and Eating Psychopathology*	Groups		P value**	Effect size ***
	NWR n=40 Median (min-max)	WR n=50 Median (min-max)		
Binge Eating Scale	6 (0-26)	11 (0-42)	**0.013**	0.55
Three-Factor Eating Questionnaire				
Restriction Subscale	13 (4-19)	9.5 (3-19)	**0.007**	-0.60
Disinhibition Subscale	4 (1-12)	6.5 (2-19)	**0.001**	0.76
Hunger Subscale	3 (0-12)	4 (1-11)	**0.004**	0.39
Body Shape Questionnaire	74 (34-192)	89.5 (36-183)	0.06	0.36
Beck Depression Inventory	8.5 (0-49)	9 (0-46)	0.43	0.16
Barratt Impulsiveness Scale				
Total Score	69 (52-80)	71 (51-90)	0.69	0.42
Motor Impulsivity	23 (16-35)	24.5 (17-35)	0.34	0.12
Attentional Impulsivity	21 (14-26)	19.5 (14-28)	0.50	-0.06
Non-Planning Impulsivity	25 (17-34)	27 (16-34)	**0.001**	0.69

*General and eating psychopathology were evaluated during the in-person interview. **Analysis performed using the Mann-Whitney U test. Significance was defined by a p value ≤ 0.05. ***Effect size was calculated using Cohen’s d statistic. According to Cohen, 0.2 could be considered a “small” effect size, 0.5 represents a “medium” effect size, and 0.8 a “large” effect size (36).

Abbreviations: max, maximum; min, minimum; NWR, no weight regain; WR, weight regain.

## DISCUSSION

The present study confirmed the association between multiple mental health domains and weight regain in patients undergoing bariatric surgery. Notably, the frequency of psychiatric comorbidities (categorical), such as current binge-eating disorder, lifetime bulimia nervosa, lifetime obsessive-compulsive disorder, and alcohol abuse/dependence, was significantly higher in participants with weight regain. Furthermore, in terms of psychopathology assessed dimensionally, disordered eating behavior and impulsivity were significantly higher in participants with postoperative weight regain. However, after a controlled analysis, only binge-eating disorder remained significantly associated with weight regain.

We found an increased prevalence of mental disorders and higher levels of psychopathology, which is consistent with other studies (8,^12^,^14^,^37^) demonstrating that the bariatric surgery population and individuals with severe obesity often have psychiatric comorbidity. In our sample, 40% of participants were using psychiatric medications. However, as we reported, there was no significant difference in weight regain comparing the groups with and without the use of psychopharmacotherapy. This is comparable to the results of a 7-year prospective study by Kalarchian and cols., which reported that 37.7% of the participants were on some form of psychiatric medication (14). Furthermore, Kalarchian and cols. found no significant difference in weight regain associated with the use of psychiatric medication (14,38). This finding deserves attention because individuals with psychiatric disorders may need closer postoperative follow-up.

We found a significantly higher lifetime prevalence of obsessive-compulsive disorder and alcohol abuse/dependence in participants with weight regain compared with those without weight regain. Considering the lack of information regarding the association between weight regain and general psychiatric comorbidity (13), this is a unique finding. Although a higher incidence of obsessive-compulsive disorder has been reported in bariatric surgery candidates (39,40) compared with individuals with severe obesity who are not candidates for bariatric surgery, our data suggests a relationship between this lifetime mental disorder and a poor surgical weight outcome. A potential explanation for this finding is that individuals with obsessive-compulsive disorder may have greater difficulty adhering to follow-up visits and recommendations because of their psychiatric impairment (40). In our study, weight regain was associated with higher rates of lifetime problematic alcohol use, with 10% showing current issues and 13.3% having a lifetime history, consistent with similar studies on patients at postoperative RYGB (41). However, the link between lifetime problematic alcohol use and weight regain remains unclear and requires further investigation. Some evidence suggests that metabolic changes following the RYGB procedure may reduce alcohol appeal for certain individuals (42). Additionally, those with a history of problematic alcohol use may draw upon coping skills from substance abuse treatment to adopt healthier habits after bariatric surgery (42), potentially explaining a proportional prevalence of current alcohol problems among our weight regain groups.

Our data indicate a significant association between lifetime diagnosis of bulimia nervosa and current binge-eating disorder with weight regain after RYGB surgery. However, the application of DSM-5 criteria to assess binge-eating spectrum disorders in this population may underestimate their prevalence due to anatomical restrictions imposed by the surgical procedure, potentially impacting the amount of food consumed during binge episodes (43). This could make the marker “loss of control while eating” more accurate for this group. In a prospective study of 748 individuals who had undergone bariatric surgery, Hilbert and cols. (43) investigated the differences in prevalence of binge-eating spectrum disorders when assessing them based on DSM-5 criteria compared with diagnoses centered on the loss of control rather than the quantity of food consumed. In the fourth year of assessment, they found a prevalence of binge-eating disorder of 0.88% using the DSM-5 criteria, compared with 1.76% when focusing on the loss of control marker. Therefore, our findings related to binge-eating disorder and bulimia nervosa based on DSM-5 criteria could be underrated. Even though the presence of binge-eating symptoms has been related to less weight loss and/or more weight regain postoperatively (18,44), various authors (13,18,45) have identified methodological flaws in the existing evidence. These authors have pointed out the need for studies using validated interviews and not only self-reported questionnaires to assess eating disorder diagnoses. Thus, the results of the present study add relevant information regarding the relationship between eating disorders and the bariatric surgery outcome of weight regain.

Overall, our findings suggest that mental disorders such as obsessive-compulsive disorder, substance use disorders, binge-eating disorder, and bulimia nervosa, which share common features of the impulsivity-compulsivity spectrum, were associated with weight regain after RYGB surgery (46). These features include emotional dysregulation or disinhibition (loss of control over eating), impulsivity, and compulsive behaviors (47-49). The interplay of these behaviors may contribute to a suboptimal outcome after bariatric surgery, including weight regain and psychosocial distress.

In the present study, postoperative weight regain was positively associated with binge-eating symptoms, eating disinhibition, and non-planning impulsivity, and negatively associated with restrictive eating habits, with moderate effect sizes. Our findings are consistent with previous studies (13,50,51) and suggest that postoperative binge eating is associated with unfavorable weight outcomes in bariatric surgery. Likewise, Dos Rodrigues in 2021 (52) and Amundsen in 2017 (53) applied the TEFQ to individuals who had undergone bariatric surgery and found that those with weight regain had a significant increase in eating disinhibition and a decrease in restrictive eating. Thus, the results of the present study revealed that binge eating, eating disinhibition, and lower food restriction were associated with weight regain. These eating behaviors may be influenced by the long-term physiological changes induced by bariatric surgery, whereas decreased hunger satiation signals, hormonal changes, and biochemical modifications may be responsible for increased eating disorder scores (26). Finally, regarding impulsivity, Marchitelli and cols. in 2022 (54) followed longitudinally 47 patients after bariatric surgery and found that non-planning impulsivity was a reliable short-term predictor of weight loss after bariatric surgery. As non-planning impulsiveness interferes with the ability to predict the consequences of one’s actions and to plan accordingly, this dimension of impulsivity (49) may have influenced adherence, leading to dysfunctional weight regain behaviors.

According to some evidence, alterations in the anatomical (gastric volume), metabolic (neurohormonal), and reward systems induced by bariatric surgery may impact the clinical presentation of psychopathology, especially disordered eating behaviors (55). First, the limitation in the gastric volume could limit the quantity of food that patients can eat postoperatively, while dysfunctional eating behaviors could change from overeating to eating smaller quantities. However, these alterations in eating habits could also induce emotional distress, owing to the loss of control over the amount of food compared with the preoperative quantity (18). Some gastrointestinal symptoms may develop *de novo* following bariatric surgery, including vomiting, dumping syndrome (resulting from rapid gastric emptying or reactive hypoglycemia), dysphagia, and chewing and spitting food (56,57). In the context of bariatric surgery procedures, it is important to consider that these gastrointestinal symptoms may be direct consequences of the surgical procedure rather than being strictly compensatory behaviors aimed at preventing weight gain (56,57). Second, after bariatric surgery, peripheral metabolic changes in gut hormones, peptides, microbiota, peripheral adiposity signals, and vagal tonus could affect general and eating psychopathology (26). These peripheral changes could influence eating psychopathology by modifying hunger homeostasis (satiety and hunger perception), which affects the amount of food consumed (26). Bariatric surgery can induce complex biological changes that interact with neurobehavioral features. However, this incipient research field requires more evidence to better elucidate these associations.

### Strengths and limitations

Some limitations of the study must be considered, including its cross-sectional design, which renders inferences and determination of long-term consequences questionable. Moreover, conclusive associations cannot be determined without an epidemiological or prospective study design, owing to the sample size and low frequency of mental disorders, especially eating disorders (18). An additional limitation of our study is that it was based on a convenience sample, which could be a source of bias. We did not include the evaluation of problematic eating issues such as dumping syndrome, dysphagia, or the act of chewing followed by spitting out food. This omission limits the scope of discussions based on our findings regarding problematic eating following bariatric surgery. Additionally, we did not collect data on participants’ maximum weight over the course of their lives, which could have potentially affected the interpretation of our weight-related outcomes. Also, some of the weight measurements applied in this study were self-reported, even though they can be adequately used in this postoperative population (58). The strengths of this study included the quality of outcome assessment with a standardized weight regain definition and validated instruments to assess mental health outcomes, including the DSM-5 criteria for eating disorder diagnoses.

### Future direction

It is still unclear whether psychiatric comorbidity could trigger weight regain or vice versa (13). Future research with a large sample cohort, longitudinal design, and standardized weight regain definition (1) is needed. Moreover, the interplay between biological factors and mental health may explain how psychopathological comorbidities could impact weight regain (13,37). Future research should investigate this association with multiple mental health approaches, to better identify or predict impulsivity domains that can affect weight outcomes (59). Furthermore, timely targeted treatments based on specific psychiatric domains that are more prone to affect weight outcomes after bariatric surgery would be helpful (60). For instance, clinical settings should consider administering psychotropic drugs to treat psychiatric comorbidity, providing specific psychological interventions to support psychopathology impairment, or providing self-guided information to promote healthier habit modifications. Due to the association between binge-eating disorders/behavior and weight regain, it is important to assess this behavior before and after bariatric surgery. A series of instruments, such as the Questionnaire on Eating and Weight Patterns-5 (QEWP-5) (61) and BES (62), may help clinicians and surgeons identify binge-eating behavior in this population. The adequate management of this condition may help overcome potential weight regain after bariatric surgery.

In conclusion, the findings of the present study indicate a high prevalence of poor mental health in patients experiencing weight regain after bariatric surgery. Thus, it is necessary to adequately assess and address mental health to improve the long-term outcomes of bariatric surgery.

## References

[1] King WC, Hinerman AS, Courcoulas AP (2020). Weight regain after bariatric surgery: a systematic literature review and comparison across studies using a large reference sample. Surg Obes Relat Dis.

[2] King WC, Hinerman AS, Belle SH, Wahed AS, Courcoulas AP (2018). Comparison of the Performance of Common Measures of Weight Regain After Bariatric Surgery for Association With Clinical Outcomes. JAMA.

[3] Majid SF, Davis MJ, Ajmal S, Podkameni D, Jain-Spangler K, Guerron AD (2022). Current state of the definition and terminology related to weight recurrence after metabolic surgery: review by the POWER Task Force of the American Society for Metabolic and Bariatric Surgery. Surg Obes Relat Dis.

[4] King WC, Hinerman AS, Courcoulas AP (2020). Weight regain after bariatric surgery: a systematic literature review and comparison across studies using a large reference sample. Surg Obes Relat Dis.

[5] Devlin MJ, King WC, Kalarchian MA, Hinerman A, Marcus MD, Yanovski SZ (2018). Eating pathology and associations with long-term changes in weight and quality of life in the longitudinal assessment of bariatric surgery study. Int J Eat Disord.

[6] Concon MM, Jimenez LS, Callejas GH, Chaim EA, Cazzo E (2019). Influence of post-Roux-en-Y gastric bypass weight recidivism on insulin resistance: a 3-year follow-up. Surg Obes Relat Dis.

[7] Jimenez LS, Mendonça Chaim FH, Mendonça Chaim FD, Utrini MP, Gestic MA, Chaim EA (2018). Impact of Weight Regain on the Evolution of Non-alcoholic Fatty Liver Disease After Roux-en-Y Gastric Bypass: a 3-Year Follow-up. Obes Surg.

[8] Romain AJ, Marleau J, Baillot A (2019). Association between physical multimorbidity, body mass index and mental health/disorders in a representative sample of people with obesity. J Epidemiol Community Health.

[9] Launders N, Hayes JF, Price G, Osborn DP (2022). Clustering of physical health multimorbidity in people with severe mental illness: An accumulated prevalence analysis of United Kingdom primary care data. PLoS Med.

[10] Flores-Dorantes MT, Díaz-López YE, Gutiérrez-Aguilar R (2020). Environment and Gene Association With Obesity and Their Impact on Neurodegenerative and Neurodevelopmental Diseases. Front Neurosci.

[11] Federico A, Spalatro AV, Giorgio I, Enrica M, Abbate Daga G, Secondo F (2019). Personality and psychopathology differences between bariatric surgery candidates, subjects with obesity not seeking surgery management, and healthy subjects. Eat Weight Disord.

[12] Dawes AJ, Maggard-Gibbons M, Maher AR, Booth MJ, Miake-Lye I, Beroes JM (2016). Mental Health Conditions Among Patients Seeking and Undergoing Bariatric Surgery: A Meta-analysis. JAMA.

[13] Mauro MFFP, Papelbaum M, Brasil MAA, Carneiro JRI, Coutinho ESF, Coutinho W (2019). Is weight regain after bariatric surgery associated with psychiatric comorbidity? A systematic review and meta-analysis. Obes Rev.

[14] Kalarchian MA, King WC, Devlin MJ, Hinerman A, Marcus MD, Yanovski SZ (2019). Mental disorders and weight change in a prospective study of bariatric surgery patients: 7 years of follow-up. Surg Obes Relat Dis.

[15] De Zwaan M, Enderle J, Wagner S, Mühlhans B, Ditzen B, Gefeller O (2011). Anxiety and depression in bariatric surgery patients: A prospective, follow-up study using structured clinical interviews. J Affect Disord.

[16] Marek RJ, Ben-Porath YS, Heinberg LJ (2016). Understanding the role of psychopathology in bariatric surgery outcomes. Obes Rev.

[17] Lavender JM, King WC, Kalarchian MA, Devlin MJ, Hinerman A, Gunstad J (2020). Examining emotion-, personality-, and reward-related dispositional tendencies in relation to eating pathology and weight change over seven years in the Longitudinal Assessment of Bariatric Surgery (LABS) study. J Psychiatr Res.

[18] Williams-Kerver GA, Steffen KJ, Mitchell JE (2019). Eating Pathology After Bariatric Surgery: an Updated Review of the Recent Literature. Curr Psychiatry Rep.

[19] Gill H, Kang S, Lee Y, Rosenblat JD, Brietzke E, Zuckerman H (2019). The long-term effect of bariatric surgery on depression and anxiety. J Affect Disord.

[20] Nicolau J, Simo R, Sanchis P, Ayala L, Fortuny R, Rivera R (2017). Effects of depressive symptoms on clinical outcomes, inflammatory markers and quality of life after a significant weight loss in a bariatric surgery sample. Nutr Hosp.

[21] Conceição E, Mitchell JE, Vaz AR, Bastos AP, Ramalho S, Silva C (2014). The presence of maladaptive eating behaviors after bariatric surgery in a cross sectional study: Importance of picking or nibbling on weight regain. Eat Behav.

[22] Kofman MD, Lent MR, Swencionis C (2010). Maladaptive eating patterns, quality of life, and weight outcomes following gastric bypass: Results of an internet survey. Obesity.

[23] Sousa P, Bastos AP, Venâncio C, Vaz AR, Brandão I, Costa JM (2014). Understanding depressive symptoms after bariatric surgery: The role of weight, eating and body image. Acta Med Port.

[24] Müller A, Hase C, Pommnitz M, de Zwaan M (2019). Depression and Suicide After Bariatric Surgery. Curr Psychiatry Rep.

[25] Haslam N, McGrath MJ, Viechtbauer W, Kuppens P (2020). Dimensions over categories: a meta-analysis of taxometric research. Psychol Med.

[26] Nicoletti CF, Delfino HBP, Ferreira FC, Pinhel MAS, Nonino CB (2019). Role of eating disorders-related polymorphisms in obesity pathophysiology. Rev Endocr Metab Disord.

[27] Brethauer SA, Kim J, el Chaar M, Papasavas P, Eisenberg D, Rogers A (2015). Standardized outcomes reporting in metabolic and bariatric surgery. Surg Obes Relat Dis.

[28] Tavares M (1997). Entrevista Clínica Estruturada para o DSM-IV Transtornos do Eixo I.

[29] American Psychiatric Association (2013). Diagnostic and statistical manual of mental disorders.

[30] Gomes-Oliveira MH, Gorenstein C, Lotufo F, Andrade LH, Wang YP (2012). Validation of the Brazilian Portuguese version of the Beck Depression Inventory-II in a community sample. Braz J Psychiatry.

[31] Malloy-Diniz LF, Mattos P, Leite WB, Abreu N, Coutinho G, Paula JJ (2010). Tradução e adaptação cultural da Barratt Impulsiveness Scale (BIS-11) para aplicação em adultos brasileiros. Rev Bras Psiquiatr.

[32] Freitas S, Lopes CS, Coutinho W, Appolinario JC (2001). Tradução e adaptação para o português da Escala de Compulsão Alimentar Periódica. Rev Bras Psiquiatr.

[33] Stunkard AJ, Messick S (1985). The three-factor eating questionnaire to measure dietary restraint, disinhibition and hunger. J Psychosom Res.

[34] Freitas S, Gorenstein C, Appolinario JC (2002). Instrumentos para a avaliação dos transtornos alimentares. Rev Bras Psiquiatr.

[35] Di Pietro M, Silveira DX (2009). Internal validity, dimensionality and performance of the Body Shape Questionnaire in a group of Brazilian college students. Braz J Psychiatry.

[36] Cohen J (1988). Statistical Power Analysis for the Behavioral Sciences.

[37] Monteleone AM, Globus I, Cascino G, Klomek AB, Latzer Y (2022). Psychopathology predicts mental but not physical bariatric surgery outcome at 3-year follow-up: a network analysis study. Eat Weight Disord.

[38] Coughlin JW, Steffen KJ, Sockalingam S, Mitchell JE (2022). Psychotropic Medications in Metabolic and Bariatric Surgery: Research Updates and Clinical Considerations. Curr Psychiatry Rep.

[39] Rutledge T, Adler S, Friedman R (2011). A prospective assessment of psychosocial factors among bariatric versus non-bariatric surgery candidates. Obes Surg.

[40] Rosik CH (2005). Psychiatric symptoms among prospective bariatric surgery patients: Rates of prevalence and their relation to social desirability, pursuit of surgery, and follow-up attendance. Obes Surg.

[41] Ivezaj V, Benoit SC, Davis J, Engel S, Lloret-Linares C, Mitchell JE (2019). Changes in Alcohol Use after Metabolic and Bariatric Surgery: Predictors and Mechanisms. Curr Psychiatry Rep.

[42] Ivezaj V, Stoeckel LE, Avena NM, Benoit SC, Conason A, Davis JF (2017). Obesity and addiction: can a complication of surgery help us understand the connection?. Obes Rev.

[43] Hilbert A, Staerk C, Strömer A, Mansfeld T, Sander J, Seyfried F (2022). Nonnormative Eating Behaviors and Eating Disorders and Their Associations With Weight Loss and Quality of Life During 6 Years Following Obesity Surgery. JAMA Netw Open.

[44] Conceicão EM, Mitchell JE, Pinto-Bastos A, Arrojado F, Brandão I, Machado PPP (2017). Stability of problematic eating behaviors and weight loss trajectories after bariatric surgery: a longitudinal observational study. Surg Obes Relat Dis.

[45] Tess BH, Maximiano-Ferreira L, Pajecki D, Wang Y (2019). Bariatric surgery and binge eating disorder: should surgeons care about it? a literature review of prevalence and assessment tools. Arq Gastroenterol.

[46] Tang DW, Fellows LK, Small DM, Dagher A (2012). Food and drug cues activate similar brain regions: a meta-analysis of functional MRI studies. Physiol Behav.

[47] Kessler RM, Hutson PH, Herman BK, Potenza MN (2016). The neurobiological basis of binge-eating disorder. Neurosci Biobehav Rev.

[48] Volkow ND, Wang GJ, Tomasi D, Baler RD (2013). Obesity and addiction: neurobiological overlaps. Obes Rev.

[49] Yeo D, Toh A, Yeo C, Low G, Yeo JZ, Aung MO (2021). The impact of impulsivity on weight loss after bariatric surgery: a systematic review. Eat Weight Disord.

[50] Fangueiro FS, França CN, Fernandez M, Ilias EJ, Colombo-Souza P (2021). Binge Eating After Bariatric Surgery in Patients Assisted by the Reference Service in a Brazilian Hospital and the Correlation with Weight Loss. Obes Surg.

[51] Freire CC, Zanella MT, Segal A, Arasaki CH, Matos MIR, Carneiro G (2021). Associations between binge eating, depressive symptoms and anxiety and weight regain after Roux-en-Y gastric bypass surgery. Eat Weight Disord.

[52] Dos Rodrigues LS, de Vasconcelos PHC, Gomes DL (2021). Weight regain and eating behavior in physically active and inactive women after 24 months of bariatric surgery. Eat Weight Disord.

[53] Amundsen T, Strømmen M, Martins C (2017). Suboptimal Weight Loss and Weight Regain after Gastric Bypass Surgery-Postoperative Status of Energy Intake, Eating Behavior, Physical Activity, and Psychometrics. Obes Surg.

[54] Marchitelli S, Ricci E, Mazza C, Roma P, Tambelli R, Casella G (2022). Obesity and Psychological Factors Associated with Weight Loss after Bariatric Surgery: A Longitudinal Study. Nutrients.

[55] Opozda M, Chur-Hansen A, Wittert G (2016). Changes in problematic and disordered eating after gastric bypass, adjustable gastric banding and vertical sleeve gastrectomy: a systematic review of pre-post studies. Obes Rev.

[56] Ivezaj V, Carr MM, Brode C, Devlin M, Heinberg LJ, Kalarchian MA (2021). Disordered eating following bariatric surgery: a review of measurement and conceptual considerations. Surg Obes Relat Dis.

[57] Kalarchian MA, King WC, Devlin MJ, White GE, Marcus MD, Garcia L (2017). Surgery-related gastrointestinal symptoms in a prospective study of bariatric surgery patients: 3-year follow-up. Surg Obes Relat Dis.

[58] Christian NJ, King WC, Yanovski SZ, Courcoulas AP, Belle SH (2013). Validity of self-reported weights following bariatric surgery. JAMA.

[59] Sarwer DB, Wadden TA, Ashare RL, Spitzer JC, McCuen-Wurst C, LaGrotte C (2021). Psychopathology, disordered eating, and impulsivity in patients seeking bariatric surgery. Surg Obes Relat Dis.

[60] Sarwer DB, Allison KC, Wadden TA, Ashare R, Spitzer JC, McCuen-Wurst C (2019). Psychopathology, disordered eating, and impulsivity as predictors of outcomes of bariatric surgery. Surg Obes Relat Dis.

[61] Moraes CEF, Mourilhe C, Veiga GVD, de Freitas SR, Luiz RR, Hay P (2021). Concurrent validity of the Brazilian Portuguese version of the Questionnaire on Eating and Weight Patterns-5 (QEWP-5) in the general population. Eat Behav.

[62] Grupski AE, Hood MM, Hall BJ, Azarbad L, Fitzpatrick SL, Corsica JA (2013). Examining the Binge Eating Scale in screening for binge eating disorder in bariatric surgery candidates. Obes Surg.

